# Vitamin D status and associated factors in recent-onset type 1 diabetic children in Iran

**DOI:** 10.1186/2251-6581-11-12

**Published:** 2012-09-03

**Authors:** Asal Ataie-Jafari, Asmah Bt Rahmat, Farzaneh Abbasi, Seng Cheong Loke, Mostafa Qorbani, Bagher Larijani

**Affiliations:** 1 Endocrinology and Metabolism Research Center/Tehran University of Medical Sciences, 5th floor, Shariati hospital, North Karegar Avenue, Tehran, Iran; 2 Department of Nutrition and Dietetics, Faculty of Medicine and Health Sciences, Universiti Putra Malaysia, 43400, Serdang, Selangor Darul Ehsan, Malaysia; 3 Growth and Development Research Center, Tehran University of Medical Sciences, 1st floor, Children Medical Center Hospital, Gharib Avenue, Terhran, Iran; 4 Institute of Gerontology (IG), Universiti Putra Malaysia, 43400, Serdang, Selangor Darul Ehsan, Malaysia

**Keywords:** Vitamin D deficiency, Type 1 diabetes, Children

## Abstract

**Background:**

In this study, the prevalence of vitamin D deficiency was assessed in a group of children and adolescent patients with recent-onset type 1 diabetes mellitus (T1DM).

**Methods:**

Fifty-three patients with age 8–18 years and duration of T1DM less than 8 weeks were recruited. A food frequency questionnaire (FFQ) was used to assess dietary vitamin D and calcium intake. Sunshine exposure was measured using a questionnaire to quantify the amount of time children spent in the sun and other sun-related habits, and a sun index score was generated. Serum 25(OH)D < 20 ng/ml was considered as vitamin D deficiency. Logistic regression was used to assess predictors of vitamin D deficiency.

**Results:**

All patients were vitamin D deficient (77%) or insufficient (23%). In a logistic regression model, it was shown that the risk of being vitamin D deficient was significantly decreased by sunlight exposure ≥ 15 minutes during the weekends versus < 15 minutes (OR: 0.06, 95% CI: 0.01–0.75; *P*=0.029). In addition, vitamin D deficiency in boys was lower than girls in this model (OR: 0.164 [95% CI: 0.02–1.11]; *P* = 0.063).

**Conclusion:**

Vitamin D deficiency is highly prevalent among children and adolescents with T1DM in Iran. Boys and children with ≥ 15 minutes sunlight exposure in weekends were less likely to be vitamin D deficient than girls and those with < 15 minutes sunlight exposure.

## Background

Type 1 diabetes mellitus (T1DM) is a complex disease characterized by autoimmune and progressive destruction of insulin secreting pancreatic β-cells. Both genetic and environmental factors are main agents participating in this autoimmune process [1,2].

The incidence is very different according to geographic variation, with a child in Finland being about 400 times more likely than a child in Venezuela to acquire the disease [3]. Season is another factor, with lower incidence rate in the summer and statistically significant peaks in the late winter and early spring months [4].

One of the environmental factors thought to be protective against the development of T1DM is early supplementation with vitamin D [5]. Vitamin D, a potent regulator of calcium and phosphate metabolism, has been shown to possess immunomodulatory properties [6]. Several studies suggest that vitamin D supplementation in early childhood decreases the risk of developing T1DM [5,6]. Moreover, there is a negative correlation between vitamin D intake of pregnant mother and the presence of islet antibodies in her child [7]. This observation suggests an immunological mechanism behind the association between vitamin D and T1DM.

The marker of vitamin D status is 25(OH)D since it is the major circulating metabolite of vitamin D [8]. Studies from different countries have shown a highly variable prevalence of vitamin D deficiency ranging from 15 to 60% among children and adolescents with T1DM [9-13]. Definition of vitamin D deficiency is controversial, however, most experts agree that 25(OH)D of <20 ng/ml is considered to be vitamin D deficiency, whereas a 25(OH)D of 21–29 ng/ml is considered to be insufficient, and a level ≥30 ng/ml is the normal value of vitamin D in both children and adults [14].

Before conducting a clinical trial of supplementing patients with T1DM, it is required to assess the existing status. In this cross-sectional study, the prevalence of vitamin D deficiency was assessed in children and adolescents with new-onset T1DM. Factors such as BMI, sex, sunlight exposure, and dietary vitamin D and calcium were assessed in relation to vitamin D deficiency. In addition, serum iPTH (intact parathyroid hormone) level was assessed to identify patients with secondary hyperparathyroidism.

## Methods

### Study design and patients

The research was approved by the Ethics Committee of Endocrinology and Metabolism Research Center (EMRC)/Tehran University of Medical Sciences. Fifty three patients with newly diagnosed T1DM were recruited from outpatient diabetes clinic in Shariati Hospital and Children Medical Center of Tehran University of Medical Sciences.

Subjects included if they were 8–18 year, consecutively diagnosed as having T1DM [15], duration of clinical disease less than 8 weeks, and without any medical co-morbidities or any other major chronic disease. Subjects were excluded from the study if they had consumed cholecalciferol, calcium, multi-vitamin or mineral supplementation, or vitamin D-fortified foods during the previous 3 months.

A 49-item semi-quantitative food frequency questionnaire (FFQ) on calcium and vitamin D intake was administered by the nutritionist. Frequencies of food intakes over the previous 3 months were reported in day, week, or month. Calcium content of foods was derived from Nutritionist III software modified for Iranian foods. Vitamin D was calculated according to the “Provisional Table on the Vitamin D Content of Foods” released by United States Department of Agriculture (USDA) [16].

Average sunshine exposure was measured using a questionnaire to quantify the amount of time children spent in the sun and other sun-related habits during past 3 months since patients’ recruitment. The questions asked about time spent outdoors, time of day when outdoors, use of sunscreen, sun protection factor (SPF), type of clothing, and the skin pigment. It was a modified version of the sun exposure questionnaire designed by Dr Glanz [17], and the questionnaire we used before [18].

Sun index score was generated as a measure combining hours spent in sun exposure during weekdays and weekends, and sun protective behaviors (i.e. clothing and sunscreen use) and patients’ skin color. Possible scores ranged from 4–14. Higher scores represented higher sunlight exposure. The sun scores were examined with respect to their relationship to serum vitamin D levels. Fasting serum 25(OH)D and intact PTH level was measured using immunoradiometric assay with the IDS kit (Boldon, Tyne and Wear, UK).

### Statistical analysis

Data was analyzed using the statistical software SPSS version 16.0 for windows (SPSS Inc., Chicago, IL). P-values < 0.05 were considered statistically significant. Logistic regression was used to assess predictors of vitamin D deficiency. Serum 25(OH)D as a dichotomous variable (1 = Vitamin D deficient, and 0 = Vitamin D insufficient) was used to examine the associations of vitamin D deficiency with sex (0 = female, 1 = male), time duration in sun exposure in weekdays (0 = < 30 minutes, 1 = ≥ 30 minutes), time duration in sun exposure during weekend (0 = <15 minutes, 1 = ≥15 minutes), color of skin (0 = brown and olive, 1 = fair), and type of clothing (0 = sun exposure limited to hand and face, 1 = sun exposure more than hand and face).

## Results

Patients were recruited at the end of summer and during autumn of Iran (September to December). The mean age of the patients was 10.3 ± 2.0 (8–14 years) and the mean duration since diagnosis of T1DM was 43 ± 15 days. Seventy-four percent of subjects were girls and 26% were boys.

Mean daily dietary calcium and vitamin D intake among patients was 931 ± 506 mg/day (range 197–2164 mg/day) and 38 ± 17 IU/day (range 13–76 IU). Daily vitamin D intakes in all patients were less than the recommended 200 IU per day for 9–13 year old children (Institute of Medicine, 1997). Calcium intake was not different between boys and girls (*P* = 0.860). Vitamin D intake was higher in boys than girls, but the difference was only borderline significant (*P* = 0.076).

**Figure 1:**
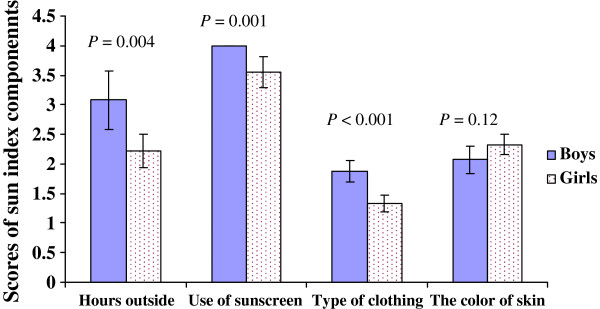
** Mean scores of different components of sun index in boys and girls.***P*-values are based on unpaired *t*-test. Bars are means, and error bars show 95% confidence interval.

Mean baseline sun index score among patients was 9.8 ± 1.5. The sun index score was significantly higher in boys than girls (11.0 ± 1.15 and 9.4 ± 1.5 respectively; *P* < 0.001). Figure 1 shows the scores of different components of sun index among girls and boys at baseline. The boys had significantly higher scores than girls for “hours outside exposed to sunlight” (3.08 ± 0.5 *vs.* 2.22 ± 0.28, *P* = 0.004), “use of sunscreen” (4.0 ± 0.0 *vs.* 3.55 ± 0.26, *P* = 0.001), and “type of clothing” (1.87 ± 0.18 *vs.* 1.33 ± 0.14, *P* < 0.001). In fact, the boys spent more time outside in the sun. In addition, their clothing was less covered than girls, and they never used sunscreen (all boys obtained score 4 for this item). The score of skin color did not differ between the boys and girls (*P* > 0.05).

Mean serum levels of 25(OH)D was 13.2 ± 6.1 ng/ml (range 4.1–27.4 ng/ml). According to these baseline values, 77% of patients were vitamin D deficient and the remaining 23% were vitamin D insufficient. No patient had sufficient level of serum 25(OH)D. There was a negative correlation between 25(OH)D concentration and iPTH level (r = −0.404, *P* = 0.003).

Serum vitamin D levels were significantly higher in boys compared to the girls (18.3 ± 7.8 and 10.8 ± 4.3 ng/ml, respectively; *P* = 0.004). In addition, there was a negative significant correlation between serum 25(OH)D and BMI (r = −0.291, *P* = 0.034). However, the difference of BMI values between vitamin D deficient and vitamin D insufficient subgroups did not reach a significant level (*P* = 0.132). Serum levels of 25(OH)D were not correlated with the age of the patients (r = − 0.148, *P* = 0.284).

Furthermore, serum 25(OH)D levels were not correlated with dietary vitamin D intake (r = 0.22, *P* = 0.349) and calcium intake (r = −0.27, *P* = 0.091). Likewise, there was no significant difference in dietary vitamin D intake among vitamin D deficient and vitamin D insufficient patients (*P* = 0.294). Calcium intake was higher in vitamin D deficient patients compared to vitamin D insufficient ones, but the difference was not significant (987 ± 531 and 729 ± 355 mg/day; *P* = 0.157).

There was a positive significant correlation between sun scores and serum levels of vitamin D (r = 0.37, *P* = 0.008). The sunlight index score was significantly lower in subjects with vitamin D deficiency compared to those with vitamin D ≥ 20 ng/ml (10.7 ± 1.4 and 9.5 ± 1.5, *P* = 0.026).

In a logistic regression model, the impact of sex and sun exposure behaviors was assessed on the likelihood of vitamin D deficiency. As shown in the Table 1, only time duration exposed to sunlight during the weekend had a statistically significant contribution to the model. In fact, the risk of being vitamin D deficient was significantly decreased by sunlight exposure ≥ 15 minutes during the weekends versus < 15 minutes (odds ratio [OR]: 0.06 [95% confidence interval [CI]: 0.01–0.75]; *P* = 0.03). The effect of sex on vitamin D status in this model was borderline significant, meaning that after adjustment for sun exposure-related behaviors, boys were less likely to be vitamin D deficient than girls (OR: 0.164 [95% CI: 0.02-1.11]; 0.10 > *P* > 0.05). The likelihood of being vitamin D deficient was not associated with the duration of sun exposure during weekdays, skin color, and type of clothing.

**Table 1:** Logistic regression model to predict vitamin D deficiency

**Model**	**Odds Ratio**	**95% CI**		***P***
**Gender**				
Boys	0.16	0.02	1.11	0.063
Girls ^a^	1			
**Hours in sun exposure in weekdays**				
≥ 30 minutes	0.82	0.11	6.11	0.845
< 30 minutes ^a^	1			
**Hours in sun exposure in weekends**				
≥ 15 minutes	0.06	0.01	0.75	0.029
< 15 minutes ^a^	1			
**Type of clothing**				
Sun exposure more than hand and face	0.28	0.03	2.28	0.232
Sun exposure less than hand and face ^a^	1			
**The color of skin**				
Fair	2.54	0.21	30.76	0.464
Olive and brown ^a^	1			

Note: ^a^ Reference group.

## Discussion

An important finding of this study was the high prevalence of vitamin D deficiency (77%) compared to those in an Australian, US, Swiss, and Italian study [9-13], demonstrating 15–60.5% deficiency using the same cut-off values to define serum vitamin D status. This range of different prevalence might be explained by differences in geographical location, the age of patients, duration since diagnosis of diabetes, and glycaemic control as suggested recently [10].

Vitamin D deficiency is a common problem even in healthy children and adolescents at a variety of different latitudes [19-23]. Few studies have assessed vitamin D status of healthy children and adolescents in some cities of Iran, demonstrating 5% vitamin D deficiency among 513 healthy 6–to 7-year-old children in Isfahan [24], and serum 25-OHD < 20 ng/ml in 78% of healthy children and adolescents (8–18 years) from Tehran [25]. Similar study in 9–12-year-old primary-school children in Tehran found that 91.7% of children had 25(OH)D < 20 ng/ml during autumn and winter [26]. The age group in the latter study was very similar to our patients’. In addition, serum samples in that study obtained during autumn and winter, and mean serum 25(OH)D was 9.5 ± 8.9 ng/ml [26]. Likewise, our baseline serum samples were drawn at the end of summer and during the autumn, and serum vitamin D levels were comparable to the aforementioned survey (13.2 ± 6.1 ng/ml). In a study in Switzerland, vitamin D deficiency rose from 60.5% to 84.1% in winter in T1DM children [11], which shows the season as an important contributor to vitamin D status. Findings from studies in Iran imply that vitamin D deficiency is a highly prevalent problem among Iranian children and adolescents, whether they are healthy or with T1DM. In accordance with previous studies [26,27], a negative correlation was observed between baseline 25(OH)D and iPTH concentrations.

Significant difference in 25(OH)D concentration between girls and boys in the present study was in agreement with previous research [25,26]. This finding could be explained by difference in sun exposure between the two genders, as a higher sun index score was shown in boys. In addition, faster growth spurt during puberty in girls and more vitamin D requirement for bones might be a further reason for the higher prevalence of vitamin D deficiency among girls than boys [25].

A significant negative correlation was found between 25(OH)D levels and BMI as previously reported [26,28,29]. Higher BMI in children and adolescents is accompanied by rapid growth of bones as they are taller than average and their bone age is slightly advanced [29]. These children need more vitamin D because of the high expenditure that would result in low blood levels of this vitamin.

Vitamin D intake in this study included only intakes from foods, since we did not enroll patients with a history of vitamin D supplementation during the previous 3 months. In addition, food sources of vitamin D are very limited, unless they are fortified. There are some vitamin D fortified foods in Iran; however they are rare and expensive. Children participating in this study did not consume any kind of foods fortified with vitamin D. As a result, low intakes of vitamin D were expected. All participants reported a daily intake of vitamin D less than the recent RDA (600 IU/day) and even lower than previous DRI (200 IU/day) [30]. Similarly, surveys in 6–7 year-old children in Isfahan [24], and 11–15 year-old girls in Tehran [31] revealed suboptimal vitamin D intake, all of them suggesting urgent need for food fortification or vitamin D supplementation in the population of Iranian children and adolescents.

Dietary vitamin D intake can be an important contributor to vitamin D status, but the current study did not find any association between vitamin D intake and serum levels of 25(OH)D. There are similar findings which show only poor correlation between vitamin D intake and serum vitamin D [32,33]. In contrast, dietary intake of vitamin D had significant effects on serum vitamin D in children in Isfahan [24]. These differences could be partly explained by differences in questionnaires or food composition tables used to estimate dietary intake, and the difference in age groups.

Low intake of calcium induces high serum PTH level and increases catabolism of 25(OH)D, therefore low intake of calcium causes decreased 25(OH)D [34]. The average daily dietary intake of calcium in this study (931 ± 506 mg/day) was almost near recommended values of 1300 mg for the 9- to 18-year- old age group. There was no correlation between calcium intake and serum 25(OH)D levels in the current trial.

Exposure to sunlight or UVB (Ultraviolet B) radiation is the primary source of vitamin D in the body [35]. In this study, both sun exposure and sun protective behaviors were included in the questionnaire to obtain a sun index score. A significant correlation between sun score and serum vitamin D levels was found. Similar results were obtained in previous studies demonstrating significant effects of sun exposure duration on serum levels of vitamin D [24]. Likewise, in a case–control study in multiple sclerosis patients in Australia [36] and a study in peri-menopausal women in Denmark [37], significant correlations was observed between various measures of self-reported sun exposure and serum 25(OH)D levels. However, correlation coefficients were low, suggesting that most of the variation in serum 25(OH)D concentrations was not explained by self-reported sun exposure. In contrast, some studies did not find significant relations between serum 25(OH)D and sunlight exposure as assessed by questionnaires [38,39]. Different results can be partly explained by different sun questionnaires, different age groups, and different regions of study.

It was found that participants with more time exposed to sun during weekend had higher levels of serum vitamin D, suggesting that as a predictor of vitamin D status in this study. Several studies have also correlated outdoor activity with vitamin D status [40-42]. UVB radiation increases vitamin D levels, as it was described earlier. However, the duration of sun exposure during weekdays was not a significant predictor of serum vitamin D in the full model of regression. It may be explained by different type of clothing during weekdays and weekends, since wearing long sleeve shirt or dress is mandatory for children in the school, but not during weekends. Effects of skin color and type of clothing on serum 25(OH)D status was not significant after controlling for sex, and other variables in the model.

## Conclusion

In summary, we observed a high prevalence of vitamin D deficiency or insufficiency among children and adolescents with T1DM. However, lack of a comparison group is the main limitation of this study, since it has been shown that vitamin D deficiency is highly prevalent in healthy children in Iran. Time duration of sunlight exposure during weekends and sex were the main predictors of serum vitamin D deficiency. Boys and children with ≥ 15 minutes exposed to sunlight in weekends were less likely to be vitamin D deficient than girls and those with < 15 minutes exposing to sunlight. Dietary vitamin D and calcium intake did not have any effect on serum vitamin D.

## Competing interests

The authors declare that they have no competing interests.

## Authors’ contributions

AA-J, design, data analysis, and interpretation, AR, design, FA, design, SCL, interpretation, MQ, interpretation, BL, design and revision. All authors read and approved the final manuscript.
